# Evaluation of Correlation between Width and Morphology of Mandibular Inferior Cortex in Digital Panoramic Radiography and Postmenopausal Osteoporosis

**Published:** 2011-03-01

**Authors:** L Khojastehpour, M Afsa, M H Dabbaghmanesh

**Affiliations:** 1 Department of Maxillofacial Radiology, Dental School, Shiraz University of Medical Sciences, Shiraz, Iran; 2 Department of Endocrinology, Shiraz University of Medical Sciences, Shiraz, Iran

**Keywords:** Osteoporosis, Postmenopause, Bone mineral density, Mandible

## Abstract

**Background:**

In osteoporotic patients, inferior mandibular cortex undergoes resorption which its manifestations can be detected on dental panoramic radiographs as a simple and available method. The aim of this study was to evaluate the correlation between width and morphology of mandibular inferior cortex in digital panoramic radiography and postmenopausal osteoporosis.

**Methods:**

Bone mineral density (BMD) of lumbar vertebrae and femural neck of 119 postmenopause women was assessed using DXA. Width [cortical index (CI)] and morphology [mandibular cortical index (MCI)] of inferior mandibular cortex were measured and the correlations between BMD and width and shape of the inferior mandibular cortex were evaluated.

**Results:**

The specificity and sensitivity in identifying women with low BMD of lumbar vertebrae by visual cortical estimation (normal or eroded) were 69.4% and 80.7% respectively. These results in identifying women with low BMD of femural neck were 67.7% and 81.5% respectively. For both sides, the threshold value that provided the highest validity (minimal false negative and false positive results) corresponded to cortical width of 4.29 mm. This threshold in lumbar vertebrae or femural neck provided a sensitivity of 81.4% (95% CI=69.1%-90.3%), specificity of 58.3% (95% CI=44.9%-70.9%), positive predictive value of 65.8% and likelihood ratio of 1.95. There were significant associations between BMD and CI and MCI.

**Conclusion:**

Postmenopausal women with thin or eroded mandibular inferior cortex may have an increased risk for low BMD or osteoporosis.

## Introduction

Osteoporosis is one of the major health problems affecting a significant number of people especially postmenopausal women; however, it can occur in men and women with underlying conditions or major risk factors associated with bone demineralization. The chief clinical manifestations are vertebral and hip fractures, although fractures can occur at any skeletal site. [1]

Nowadays, because of higher life expectancy, a larger number of osteoporotic patients may visit dentists for oral care or treatment. Annually, a large number of panoramic radiographs are taken for diagnosis and treatment of dental diseases such as dental caries and periodontal problems.[2] There are evidences in the published work suggesting that dental radiographic findings might be indicators of osteoporosis. [3][4][5][6][7][8] The mandibular cortical bone undergoes a resorptive activity in osteoporotic subjects and its manifestations can be detected on dental panoramic radiographs. [2] These evidences reveal that observing thinning of the mandibular inferior cortex and changes in the morphology of the endosteal margin of the cortex can be utilized as simple and available tools in detection of probable low bone mineral density (BMD), and referring high-risk patients for further assessment by bone densitometry to prevent the development of the disease or itscomplications. [6] There are studies that have reported significant correlations between BMD and thickness of inferior mandibular cortex in mental foramen region [Mental Index (MI)] on dental panoramic radiographs. [4][8][9][10][11] On the other hand, one of the most established indices for qualitative assessment (morphology) of inferior mandibular cortex is Mandibular Cortical Index (MCI), according to Klemetti [3] classification as follows:

C1: The endosteal margin of the cortex is even and sharp on both sides, C2: The endosteal margin of the cortex shows semilunar defects (lacunar resorption) and/or seems to form endosteal cortical residues on one or both sides and C3: The cortical layer forms heavy endosteal cortical residues and is clearly porous.

The aim of this study was to evaluate the correlation between width and morphology of mandibular inferior cortex on digital panoramic radiographs and postmenopausal osteoporosis.

## Materials and Methods 

Between 1387 and 1388, among healthy nonsmoker postmenopause Iranian women who referred to the center of BMD assessment of Nemazee Hospital, 119 subjects who participated voluntarily and met the study criteria were included in this study. The exclusion criteria were: 1) Unknown precise medical history; 2) Tobacco or alcohol use; 3) Metabolic bone diseases (hyperparathyroidism, hypoparathyroidism, Paget’s disease, osteomalacia, renal osteodystrophy and osteogenesis imperfecta); 4) Cancer with bone metastasis; 5) Diabetes; 6) Major renal impairment; 7) The use of medications that affect bone metabolism (other than estrogen); 8) Bone destructive lesions in the jaw bones (such as malignant tumors or osteomyelitis) and 9) Vertebral or non-vertebral osteoporotic fractures. At the time of BMD assessment, the study was approved by local Ethics Committee and, at the time of BMD assessment, all of the subjects were asked to give informed consent to a dental panoramic examination for oral care.

BMD at the lumbar vertebrae (L2-L4) and neck of the femur were determined using dual energy x-ray Absorptiometry (DXA, LUNAR DPX IQ). Low BMD was defined as a BMD T-score of -1 or less and osteoporosis was defined as a BMD T-score of -2.5 or less, according to the World Health Organization (WHO classification).

Dental panoramic radiographs were obtained at maximum 2 weeks after BMD assessment. Digital panoramic radiograph was prepared for each participate using Digora PCT Sorodex equipments and Pro-max panoramic X-ray unit (Planmeca, Helsinki, Finland) with the same exposure parameter (Kvp=68 and mA=9).The position of head was standardized as much as possible. Mandibular inferior cortical width and morphology (erosion) were estimated as indicators of alterations of mandible by two observers (an oral radiologist and a post graduate student of oral radiology). Each observer performed qualitative measurements (morphology) twice with two weeks interval.

The mandibular cortical width was measured bilaterally on the radiographs at the site of the mental foramen. A line parallel to the long axis of the mandible and tangential to the inferior border of the mandible was drawn. A line perpendicular to this tangent intersecting the inferior border of the mental foramen was constructed, along which the mandibular cortical width was measured. For measuring the width of the cortex, we used AUTO CAD 2009 software. The mean cortical width of both sides was used.

Mandibular cortical shape on the dental panoramic radiographs was determined by observing the images on the computer monitor, with the magnification factor of 1, using DIGORA software. Mandibular cortical index was observed distally from the mental foramen bilaterally and was categorized into one of three groups according to the method of Klemetti et al. as follows:

Normal cortex (I): The endosteal margin of the cortex is sharp and even on both sides, Mildly to moderately eroded cortex (II): The endosteal margin shows semilunar defects (lacunar resorption) or appears to form endosteal cortical residues and severely eroded cortex (III): The cortical layer forms heavy endosteal cortical residues and is clearly porous

Predictive Analysis Software (PASW, (SPSS version 18)) was used to analyze the results of this study.

Subjects were divided into 2 groups based on the assessments of the mandibular inferior cortex on panoramic views: Of normal cortex and of eroded cortex. The subjects were also divided into 2 groups according to the BMD of femural and spinal BMD: Patients of normal BMD and patients of low BMD (osteopenic and osteoporotic). The specificity and sensitivity of low BMD in subjects with an eroded cortex were calculated in dichotomous 2x2 tables. Receiver-operating curve analysis was used to determine the optimal cut-off threshold of cortical width for identification of spinal or femural osteoporosis. The second time measurements of MCI of the observer with higher intra observer agreement were used for statistical analysis.

In our study, intra-and interobserver agreements for scoring KI were assessed by calculation of the Cohen Kappa statistics. Interpretation of the Kappa statistics was based on the guidelines of Landis and Koch: Less than 0.00 (poor), 0.00-0.20 (slight), 0.21-0.40 (fair), 0.41-0.60 (moderate), 0.61-0.80 (substantial) and 0.81-1.00 (almost perfect).

One way ANOVA test was used to evaluate the correlation between age and BMD and cortical shape, the correlation between cortical width and cortical shape and also the relationship between BMD and cortical width. Pearson’s correlation coefficient was calculated to evaluate the association between cortical width and age. To evaluate the relationship between cortical shape and femural and vertebral BMD, Chi-Square test was used.

## Results

One hundred and nineteen subjects were included in this study. The subjects aged 39-91 years old with the mean age of 55.85±8.22 Years. Among the subjects, 50 had normal BMD, and 50 were osteoporotic in lumbar vertebrae or femural neck and 19 had osteopenia (Table 1). The mean age of osteoporotic and osteopenic groups had no statistically significant difference, but this difference was significant between the normal BMD and osteopenic/osteoporotic groups. The results were the same for vertebral and femoral BMD groups.

**Table 1:** Distribution of subjects in different groups of BMD, mean age and mean cortical width

	**BMD of lumbar spine **			**BMD of femural neck **			** Total **
	**normal **	**osteopenic **	**osteoporotic **	**normal **	**osteopenic **	**osteoporotic **	
Number (%)	62(52.1%)	16(13.4%)	41(34.5%)	65(54.6%)	32(26.9%)	22(18.5%)	119
Mean age	52.35	57.31	59.09	52.11	57.97	60.59	55.85
Mean cortical Width (mm)	4.35	3.57	3.34	4.29	3.36	3.52	3.90

Age was significantly correlated with cortical width (r=-0.247, p< 0.001) and cortical shape of the mandible (p<0.001). There was a significant correlation between cortical width and cortical shape of the mandible (p<0.001). For both vertebral and femural BMD, there was a statistically significant difference between the cortical width of normal and osteopenic/osteoporotic groups, however this difference was not significant between the osteopenic and osteoporotic groups (Table 2). BMD was significantly correlated with cortical shape in both lumbar vertebrae and femural neck regions (p<0.001).

**Table 2:** Distribution of subjects according to cortical shape and their mean age and mean cortical width

		**BMD of lumbar spine**			**BMD of femural neck**			Mean age	Mean cortical width(mm)
**Cortical shape**	**number**	**normal**	**osteopenic**	**osteoporotic**	**normal**	**osteopenic**	**osteoporotic**		
normal	54	43(79.6%)	4(7.4%)	7(13.7%)	44(81.5%)	7(13%)	3(5.6%)	51.19	4.37
Moderately eroded	51	17(33.3%)	8(15.7%)	26(51%)	18(35.3%)	18(35.3%)	15(29.4%)	57.66	3.81
Severe eroded	14	2(14.3%)	4(28.6%0	8(57.1%)	3(21.4%)	7(50.7%)	4(28.6%)	62.64	2.41

**Fig. 1:**
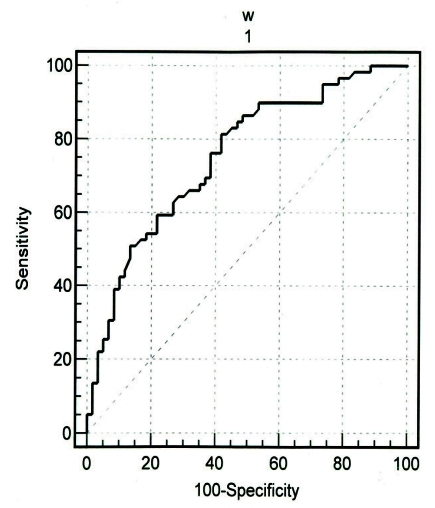
Roc curve showing the diagnostic validity of the precise cortical width in identifying postmenopausal women with low bone mineral densities of the lumbar spine or femural neck.

The specificity and sensitivity in identifying women with low BMD of lumbar vertebrae by visual cortical estimation (normal or eroded) were 69.4% and 80.7% respectively. These results in identifying women with low BMD of femural neck were 67.7% and 81.5% respectively (Table 3). The Kappa index and intraobserver agreement for the first observer in MCI classification were o.637% and 78.1%, respectively. These results for the second one were 0.564% and 73.1%, respectively. For the first time of measurement, Kappa index and interobserver agreement were 0.670% and 79.8 %. These calculations for the second time were 0.436% and 65.6%, respectively (Table 4) (Figure 1).

**Table 3:** Specificity, sensitivity and PPV of MI and MCI in identifying low BMD in lumbar Vertebrae and femural neck regions

**Index**	**Lumbar vertebrae**			**Femural neck**		
	**sensitivity**	**specificity**	**PPV**	**sensitivity**	**specificity**	**PPV**
MI	82.5%	58.1%	64.4%	81.5%	55.4%	60.3%
MCI	80.7%	69.4%	70.7%	81.5%	67.7%	67.7%

**Table 4:** Iner- and intra observer agreements

	**First time **	**Second time **	**First observer **	**Second observer **
**Kappa index and inter observer agreement **	0.67 79.8%	0.43 65.6%	—	—
**Kappa index and intra observer agreement **	—	—	0.63 78.1%	0.56 73.1

When we used the cortical width measurements, the area under the ROC curve in identifying women with low BMD of the lumbar vertebrae and femural neck were 0.764 [standard error (SE), 0.043; 95% confidence interval (CI), 0.678-0.837] and 0.737 (SE=0.045; 95% CI, 0.649-0.814) respectively when a T-score less than -1 was used as the cut-off point. For both sides, the threshold value that provided the highest validity (minimal false negative and false positive results) corresponded to cortical width of 4.29 mm. This threshold in lumbar vertebrae or femural neck provided a sensitivity of 81.4% (95% CI=69.1%-90.3%), specificity of 58.3% (95% CI=44.9%-70.9%), positive predictive value of 65.8% and likelihood ratio of 1.95 (Figure 1).

## Discussion

The reliability of KI as a predictor of osteoporosis has been proved by the following studies: Nakamato et al. [12] and Taguchi et al. [13][14] In our study, Kappa indices for intra-and inter observer agreements in MCI classification were higher than 0.41 that based on the concept of Fleiss [15] were considered to have sufficient agreement which is in accordance with the results of Nakamato and Taguchi.

The measurement of mandibular cortical thickness at the mental, gonial or antegonial regions are included in panoramic radiographic assessment. Studies have shown that measurement at mental foramina region has the best intra-or inter observer agreement and sufficient specificity for the prediction of osteoporosis. [16][17] So, we performed cortical thickness measurement at mental foramen region.

Our results showed that the mean age of subjects was higher in the osteopenic/osteoporotic groups than the normal group. Also, age was shown to have a significant correlation with the MCI classification. As age increased, the likelihood of the subjects belonging to C2 and C3 categories increased as well. These results are in agreement with the results of Knezovic-Zlataric et al. [18] and Gulsahi et al. [4] Also, in the present study, it was shown that as age increased, there was a decreasing rate in cortical width. This is in agreement with the results of Taguchi et al. [19]

We have found significant correlation between vertebral and femural BMD and the mandibular cortical width (MCW). The mean cortical widths of the osteopenic/osteoporotic groups were lower than that of the normal group. These correlations are similar to those found by Taguchi et al. [19] Konstantinos et al. demonstrated that a decrease in MCW by 1 mm increases the likelihood of osteopenia or osteoporosis to 43%. [7] However, some studies have failed to prove that a significant difference exists in mandibular cortical thickness between osteoporotic and control subjects. [5]

In our study, threshold values for cortical widths were 4.29 mm in the femural neck and lumbar spine when T-score less than -1 in the lumbar vertebrae or femural neck were considered as low BMD. Lee et al., [10] provided the highest validity. When subjects were classified based on cortical width, the areas under the ROC curve in identifying women with low BMD of the lumbar vertebrae and femural neck were 0.764 and 0.737, respectively, which corresponded to moderate accuracy. [20] These results were similar to those of Lee et al. [10] and Devlin et al. [16] At the cortical width of 4.29 mm, the overall sensitivity, specificity and positive predictive value in identifying low skeletal BMD were 81.4%, 58.3% and 65.8% respectively. Comparing with cortical shape, these results show that the specificity of cortical width is lower than that of cortical shape in identifying low BMD which indicates that cortical shape can identify more subjects with normal BMD.

When BMD of the lumbar vertebrae was used as standard, the sensitivity and specificity of KI score in identifying women with low BMD were 80.7% and 69.4% respectively with positive predictive value of 70.7%. These results when BMD of femural neck was used as standard were 81.5%, 67.7%, and 67.7%. These results show that approximately 70% (ppv) of subjects with eroded inferior cortex of mandible were of low skeletal BMD. The sensitivity shows that about 20% of subjects with low skeletal BMD are missed if MCI used as screening tool. In both sites, the specificity of cortical shape in identifying low BMD was approximately 70% which shows that cortical shape as a screening tool can identify 70% of subjects with normal BMD correctly.

Since the vertical magnification differs among panoramic machines, [21] our data for mandibular cortical width can not directly be applied to other equipment. Regarding the limitations of the present study, it is important to note that the dental condition of the patients was not taken into consideration as a variable. Some investigators revealed that the status of the mandibular dentition may exert an influence on the mandibular indices. [4] However, there are published results that show no association of dental status with mandibular indices on panoramic radiographs. [17][22]

In this study, we used panoramic radiographs obtained in a digital manner. For assessing and measuring the width and shape of mandibular inferior cortex, the observers visualized the images on the computer monitor using the software to manipulate the images. In spite of this, intra-and inter observer agreements were not higher than the other studies used traditional images for observing and measurements. [12][13][14] This may imply that using digital radiography and associated software would not improve the reproducibility of the measurements or the accuracy of the diagnosis.

Another limitation of this study is that all subjects were the postmenopausal women which are not the appropriate representative of the normal population. Although these women are most prone to osteoporosis, further studies are needed to validate the methods on male and female populations of different age groups.
